# Comparative transcription analysis of different Antirrhinum phyllotaxy nodes identifies major signal networks involved in vegetative-reproductive transition

**DOI:** 10.1371/journal.pone.0178424

**Published:** 2017-06-01

**Authors:** Dongliang Wang, Geyang Cao, Peng Fang, Lin Xia, Beijiu Cheng

**Affiliations:** 1 School of Horticulture, Anhui Agricultural University, Hefei, China; 2 Key Laboratory of Crop Biology of Anhui Province, Anhui Agricultural University, Hefei, China; Lund University, SWEDEN

## Abstract

Vegetative-reproductive phase change is an indispensable event which guarantees several aspects of successful meristem behaviour and organ development. *Antirrhinum majus* undergoes drastic changes of shoot architecture during the phase change, including phyllotactic change and leaf type alteration from opposite decussate to spiral. However, the regulation mechanism in both of phyllotactic morphology changes is still unclear. Here, the Solexa/Illumina RNA-seq high-throughput sequencing was used to evaluate the global changes of transcriptome levels among four node regions during phyllotactic development. More than 86,315,782 high quality reads were sequenced and assembled into 58,509 unigenes. These differentially expressed genes (DEGs) were classified into 118 pathways described in the KEGG database. Based on the heat-map analysis, a large number of DEGs were overwhelmingly distributed in the hormone signal pathway as well as the carbohydrate biosynthesis and metabolism. The quantitative real time (qRT)-PCR results indicated that most of DEGs were highly up-regulated in the swapping regions of phyllotactic morphology. Moreover, transcriptions factors (TFs) with high transcripts were also identified, controlling the phyllotactic morphology by the regulation of hormone and sugar-metabolism signal pathways. A number of DEGs did not align with any databases and might be novel genes involved in the phyllotactic development. These genes will serve as an invaluable genetic resource for understanding the molecular mechanism of the phyllotactic development.

## Introduction

The life cycle of higher plants develops sequentially through several distinct developmental stages: embryogenetic, vegetative, and reproductive stages. The transition from vegetative to reproductive phase is a critical developmental process, which is accompanied by the production of novel reproductive structures, such as flowers or cones [1,2]. In the vegetative phase, the shoot apical meristem (SAM) produces leaf primordia in regular phyllotaxy, and transforms to the inflorescence meristem when plant enters reproductive growth [3]. *Antirrhinum majus* displays the opposite decussate phyllotaxis during the vegetative phase, followed by a spiral phyllotaxy with the onset of the reproductive phase [4,5].

Although the mechanism underlying the vegetative-reproductive transition remain largely unknown, several studies using heterochronic mutants have revealed that the regulation of stem cell differentiation located on SAM plays a significant role in the phase change. The activity and growth of SAM determine overall plant architecture, dynamically controlled by complex and overlapping signaling networks, including the CLAVATA (CLV)-WUSCHEL (WUS) negative feedback loop, KNOX pathways, and in part through their effects on hormone signaling [6–8]. Within the established SAM, WUS positively promotes the expression of *CLV3* in the organizing center. CLV3, as a secreted peptide, is perceived by CLV1, CLV2, and RPK2/TOADSTOOL receptors, which in turn represses *WUS* expression [8–12]. In parallel to the local activity of the WUS-CLV feedback system, the Class I KNOTTED1-like homeobox (KNOXI) gene is required throughout SAM to inhibit cell differentiation. Mutations in KNOX genes lead to smaller or terminated meristems [13,14]. Moreover, KNOXI is also closely connected with plant hormone signaling. It promotes cytokinin accumulation by transcriptional activation of the cytokinin biosynthesis, which in turn activates KNOX genes, forming an apparent positive feedback loop [15–17]. Based on the mathematical model and experimental verification, an auxin-driven polarized transport by PIN-formed1 (PIN1) regulates the plant organ size and positioning [18–25]. High concentrations of auxin within SAM are associated with regions of leaf initiation, whereas the feedback between auxin and PIN-formed1 (PIN1) leads to the formation of local auxin concentration maxima [20–25].

Genetic analysis showed that several transcription factors (TFs) also participate into the regulation of the vegetative-reproductive transition such as APETALA2 (AP2), MADS-box and Homeobox [26–30]. AP2 promotes SAM maintenance by promoting WUS expression [30,31]. MADS-box TF family was included in transition from vegetative to reproductive phase and in the inner rounds of flower organ determination [26,27]. In *Antirrhinum majus*, the undifferentiated stem cells are maintained by the homeobox gene ROSULATA (ROA) [32], expressed in the quiescent zone of SAM. A meristematic identity of SAM is also related to the expression of HIRZINA (HIRZ) and INVAGINATA (INA), belonging to the KNOTTED family [33,34]. Until now, the mechanism responsible for vegetative-reproductive transition this extremely is still one of the most fascinating enigmas in plant biology.

Accumulated evidences indicated that the establishment of Antirrhinum phyllotactic morphology is determined by the vegetative-reproductive transition of SAM [4,5,35]. To obtain a comprehensive overview of transcripts, the Illumina sequencing was devoted to generating the transcriptomes of different phyllotactic nodes. By contrast, a general transcriptomic analysis showed enrichment and overexpression of differently expressed genes (DEGs) involved in hormone signal pathway and carbohydrate metabolism. Several TFs also participate the regulation of vegetative-reproductive transition. Furthermore, the quantitative real time (qRT)-PCR was performed to analyze the transcript profiles of DEGs in different phyllotactic nodes. Our results will be benefit for identifying some valuable genes and elaborating the regulation mechanism of phyllotaxy fate.

## Materials and methods

### Plant materials

The surface-sterilized seeds of wild-type *Antirrhinum majus* (stock JI-98) were grown on Murashige-Skoog (MS) plates with a controlled condition (22°C, 16 h light /8 h dark) in growth chambers. Seedlings with two internodes in appropriate developmental stages were transplanted to the pots (5 L volume) in a greenhouse (16 h/8 h, light/dark; 25°C). Plants were watered as required with an automatic drip irrigation system. When plants flowered and at least one flower on the primary stem had opened, the enlarged SAM and the phyllotactic pattern were detected for histological identification. Based on the switching charateristics of phyllotactic morphology, four development stages (S1, S2, S3 and S4) were set up between vegetative and reproductive phase. The nodes of four stages were sampled from different branches in the same Antirrhinum plant. Thirty nodes in each stage were collected from three independent plants. Three replicates were performed in each stage.

### Histological analysis

For paraffin sectioning, Antirrhinum samples were fixed overnight at 4°C in FAA (formalin: glacial acetic acid: 95% ethanol; 1:1:18), and dehydrated in a graded ethanol series. Following substitution with xylene, the samples were embedded in Paraplast Plus (Oxford Labware, St. Louis, MO) and sectioned in 8 μm thicknesses using a rotary microtome (Leitz). After stained with safranin-fast green, sections were observed using a microscope (NikonE200; Olympus Optical Co., Tokyo) equipped with Nomarski differential interference contrast optics.

### RNA extraction, library construction and RNA-seq

Total RNA was extracted from Antirrhinum nodes with a Picopure RNA isolation kit (Life Technologies) according to the manufacturer’s instruction. RNA quality was monitored using a RNA 6000 Nano chip on a BioAnalyzer 2100 (Agilent Technologies). The SMART^TM^ PCR cDNA Synthesis Kit (BD Biosciences Clontech, United States) was used for cDNA synthesis from 1 μg of poly (A^+^) RNA according to the manufacture instruction. An equal amount of total RNA from each of different preparations was pooled and used for library preparations. The first strand cDNA was amplified with PCR primer provided in the SMART^TM^ PCR cDNA Synthesis Kit. The PCR mixture (50 μL) contained 1 × Advantage 2 PCR reaction buffer, 0.3 mmol L^-1^ primers, 1 × Advantage 2 Polymerize mix, 200 mmol L^-1^ dNTPs and 5 ng first-strand cDNA. Sixteen PCR cycles (95°C for 7 s, 65°C for 20 s, and 72°C for 3 min) were performed. Four mRNA-seq libraries from different phyllotactic patterns were constructed and sequenced from both 5′ and 3′ ends by Novogene Bioinformatics Technology Co., Ltd (Beijing, China) on an Illumina HiSeq 2000 platform.

### Sequence assembly and functional annotation

Image deconvolution and Q-value were calculated in the Illumina data processing pipeline (version 1.6). Before assembly, the raw reads were filtered by discarding adaptor sequences, empty reads, low-quality reads, and shorter reads (shorter than 100 bp) using the SeqClean program (http://compbio.dfci.harvard.edu/tgi/). All clean reads were assembled twice into contigs using the Trinity program [36]. To annotate isotigs and singletons, the non-redundant sequences were searched against the protein databases (https://www.ncbi.nlm.nih.gov/protein/) obtained from NCBI with a search threshold of *E*-value cut-off 10^−5^. Based on the Blast X top hit genes, these unigenes were annotated using the BLASTx alignment (*E*-value = 10^−5^) against NCBI non-redundant (nr) database (https://blast.ncbi.nlm.nih.gov/), UniProt/Swiss-Prot (http://sparql.uniprot.org/uniprot), Kyoto Encyclopedia of Genes and Genomes (KEGG, http://www.genome.jp/kegg/), Cluster of Orthologous Groups of proteins (COG, https://www.ncbi.nlm.nih.gov/COG/) and Gene Ontology (GO) databases (http://www.geneontology.org/). GO annotations were performed by the Blast2GO program [37]. The Web Gene Ontology Annotation Plot (WEGO, http://wego.genomics.org.cn/cgi-bin/wego/index.pl) was implemented to run the GO functional classifications for all sequences [38]. The proteins with the highest sequence similarity identified in this study were retrieved and their functional annotations were determined.

### Quantitative real-time PCR analysis

The selected differentially expressed transcript factors were confirmed through qRT-PCR using ABI 7300 Real-Time PCR Detection System (Applied Biosystems, Foster City, CA, USA) with SYBR Premix Ex TaqTM II (Takara, Tokyo, Japan). Total RNA (1 μg) was converted into single-stranded cDNA using a PrimeScript^®^ Reagent Kit with gDNA Eraser (Takara, Dalian, China). The quantitative reaction was carried out in a 20 μg mixture with 10 μg FastStart Universal SYBR Green Master Rox (Roche), 6 μg milli-Q water, 1 μg primers (10 μg mol/L), and 2 μg cDNA template. The reaction was performed in an Applied Biosystems 7500 real-time PCR system (Applied Biosystems, CA, USA) at 50°C for 2 min, 95°C for 10 min, and 40 cycles at 95°C for 10 s, and 60°C for 1 min. A threshold of 0.1 was manually defined to obtain a threshold cycle (CT) value, which is required for the SYBR Green fluorescent signal (ΔRn) to cross the threshold value. Averages and standard errors (SEs) for CT values were calculated for each gene of interest based on three replications with cDNA derived from three different RNA extractions. PCR efficiencies of all primers were calculated using dilution curves with seven dilution points, a 3-fold dilution, and the equation E = [10^(-1/slope)^]^-1^. To compare data from different PCR reactions and cDNA templates, CT values for all genes were normalized to the CT values of *β*-actin (KJ018763) (CTT) for each qRT-PCR. Primer sequences were listed in S1 Table. A dissociation (melting) curve was run for each gene at the end of the amplification reaction to ensure that the DNA was amplified in the PCR reaction.

### Statistical analysis

All data were expressed as means ± the standard error (SE) and subjected to statistical analysis with the SPSS Version 20.0 software (SPSS Inc., USA). Significant differences between means were determined using One-way ANOVA followed by Tukey post hoc comparison tests at p < 0.05. Princomp procedure SAS 9.1 (SAS Institute, Cary, NC) was used for principal component analysis. The first two principal components, which explain nearly 90% of the total variation were extracted from the covariance matrix.

## Results

### Characteristics of Antirrhinum phyllotactic development

**Fig 1:**
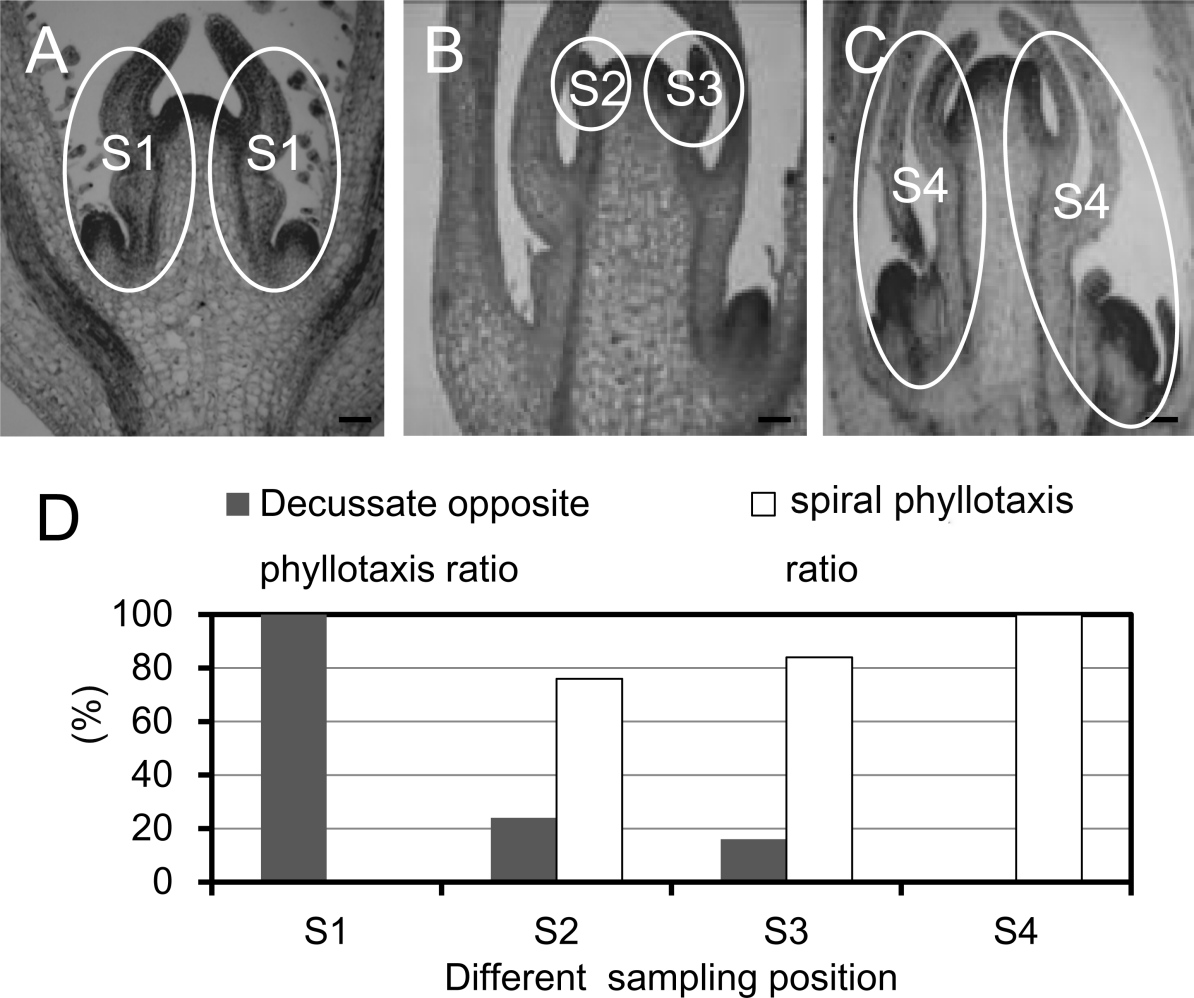
Longitudinal sections of caulis and characteristics of phyllotactic patterns. A, characteristics of opposite decussate phyllotaxy; B, swapping between opposite decussate and spiral phyllotaxy; C, characteristics of spiral phyllotaxy; D, the ratio of opposite decussate and spiral phyllotaxy in different sampling positions.

*Antirrhinum majus* displays the opposite decussate phyllotaxis during the vegetative phase, followed by a spiral phyllotaxy with the onset of the reproductive phase [4,5]. To ascertain the vegetative or reproductive phase change, the anatomical morphology of leaf primordium was shown on different Antirrhinum phyllotaxis by histological analysis. In the S1 position, all phyllotactic patterns were the opposite decussate leaf primordia observed among 156 nodes from 26 plants (Fig 1A). A different swapping from the opposite decussate to spiral leaf primordia was observed in the S2 and S3 positions (Fig 1B). In the S4 position, all growth cones developed into spiral leaf primordial (Fig 1C). 75.6% and 84% of spiral leaf primordia were respectively measured in S2 and S3 (Fig 1D). We found that the phyllotactic swapping was generated between S2 and S3. To investigate the differentially expressed genes (DEGs) involved in vegetative-reproductive transmit, four samples (named S1, S2, S3 and S4, respectively) from different Antirrhinum nodes were selected for transcriptome analysis.

### Sequencing, assembly and annotation of Antirrhinum transcriptome

Four RNA-seq libraries were constructed using total RNA from S1 to S4. 21,764,938, 21,517,401, 19,929,468 and 23,103,975 raw reads were generated, respectively. After removing low quality and short sequences, over 2.21 G of 49 nt single-end read data were produced with a Q20 percentage of about 98.72% (Table 1). The percentage of unassigned base “N” was 0.00% and the average GC content was around 45%. The trinity package assembled 58,509 unigenes with a mean size of 868 bp. The size distribution of unigenes was shown in Table 1 and S2 Table.

**Table 1:** Summary of the sequencing and assembly results.

**Sequencing information**										
**Sample ID**	**ReadSum**		**BaseSum**	**GC (%)**		**Q20%**	**N (%)**	**CycleQ20%**		**Q30%**
***A*.*majus*-S1**	21764938		4395721110	45.51		98.73	0	100		93.55
***A*.*majus*-S2**	21517401		4345315700	45.88		98.72	0	100		93.51
***A*.*majus*-S3**	19929468		4025263788	45.43		98.71	0	100		93.48
***A*.*majus*-S4**	23103975		4665368237	45.9		98.71	0	100		93.46
**Size distribution of unigenes**										
**Length range**		**Contigs**			**Transcripts**				**Unigenes**	
**200–300**		5729058 (99.09%)			19143 (14.62%)				15886 (27.15%)	
**300–500**		19481 (0.34%)			20198 (15.43%)				14730 (25.18%)	
**500–1000**		16340 (0.28%)			24557 (18.76%)				12296 (21.02%)	
**1000–2000**		10946 (0.19%)			34069 (26.02%)				9360 (16%)	
**2000+**		5641 (0.10%)			32948 (25.17%)				6237 (10.66%)	
**Total number**		5781466			130915				58509	
**Total length**		277611832			185010498				50793970	
**N50 length**		47			2224				1530	
**Mean length**		48.02			1413.21				868.14	

**Fig 2:**
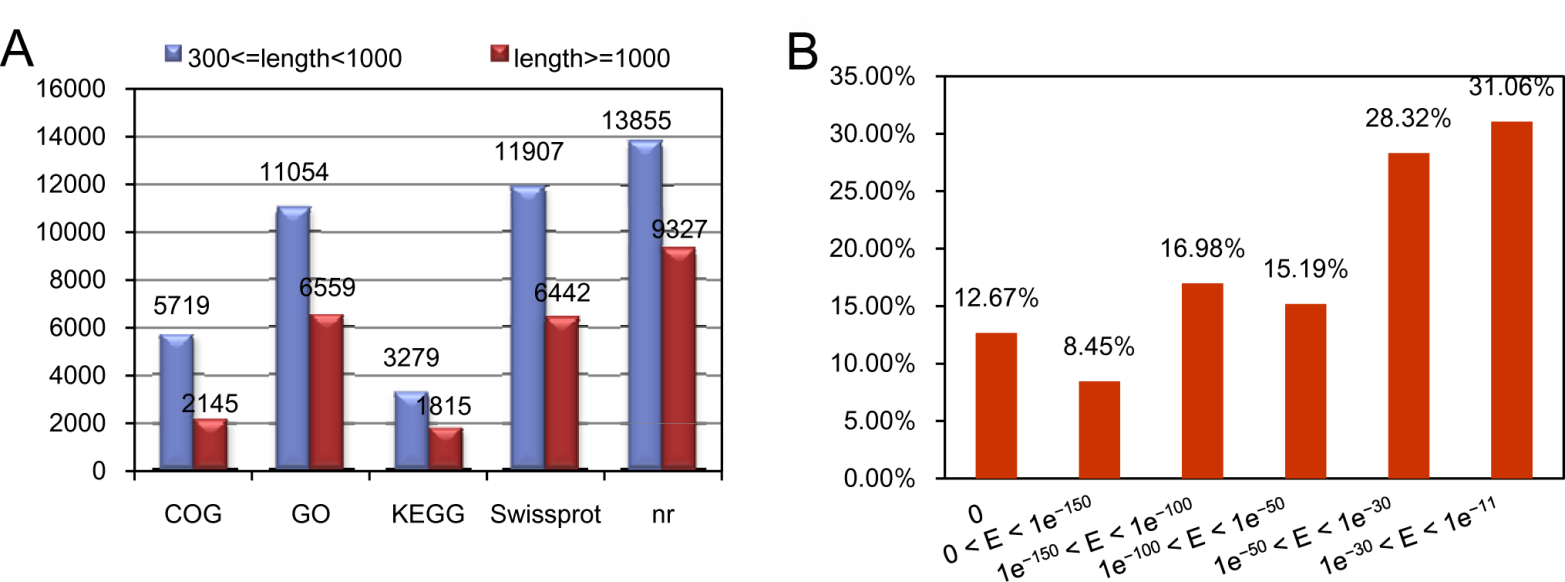
Homology analysis of the assembled unigenes against different databases. (A), the number distribution of unigenes using different annotation with a cut-off *E*-value of 1 e^-5^; (B), *E*-value distribution of top hits for each unigene.

All unigenes were aligned against the different databases with an *E*-value threshold of 1e^-5^. A total of 26,468 unigenes were annotated, while the remainder could not be done due to lacking genetic data. The number of unique best Blast X hits from non-redundant (nr), UniProt, KEGG, and COG databases was 26,535, 20,474, 5,817, 19,933 and 8,577, respectively (Fig 2A). The identity distribution was analyzed according to *E*-value of top hits in the nr database (Fig 2B). To facilitate Antirrhinum transcripts into putative function groups, GO terms were assigned using Blast2GO. About 56 functional groups including 19,933 unigenes were classified in “molecular function”, “biological process” and “cellular component” categories (S1 Fig). The molecular function was mainly made up of “catalytic activity (43.25%)” and “binding activity (41.66%)”. For the cellular components, the vast majority were related to “cell” (23.16%), “cell part (23.16%)”, “organelle (17.83%)” and “membrane (10.98%)”. Among the biological process, the majority of sequences were grouped into “cellular processes” (19.90%) and “metabolic processes” (19.54%).

### Identification of differentially expressed genes (DEGs)

**Fig 3:**
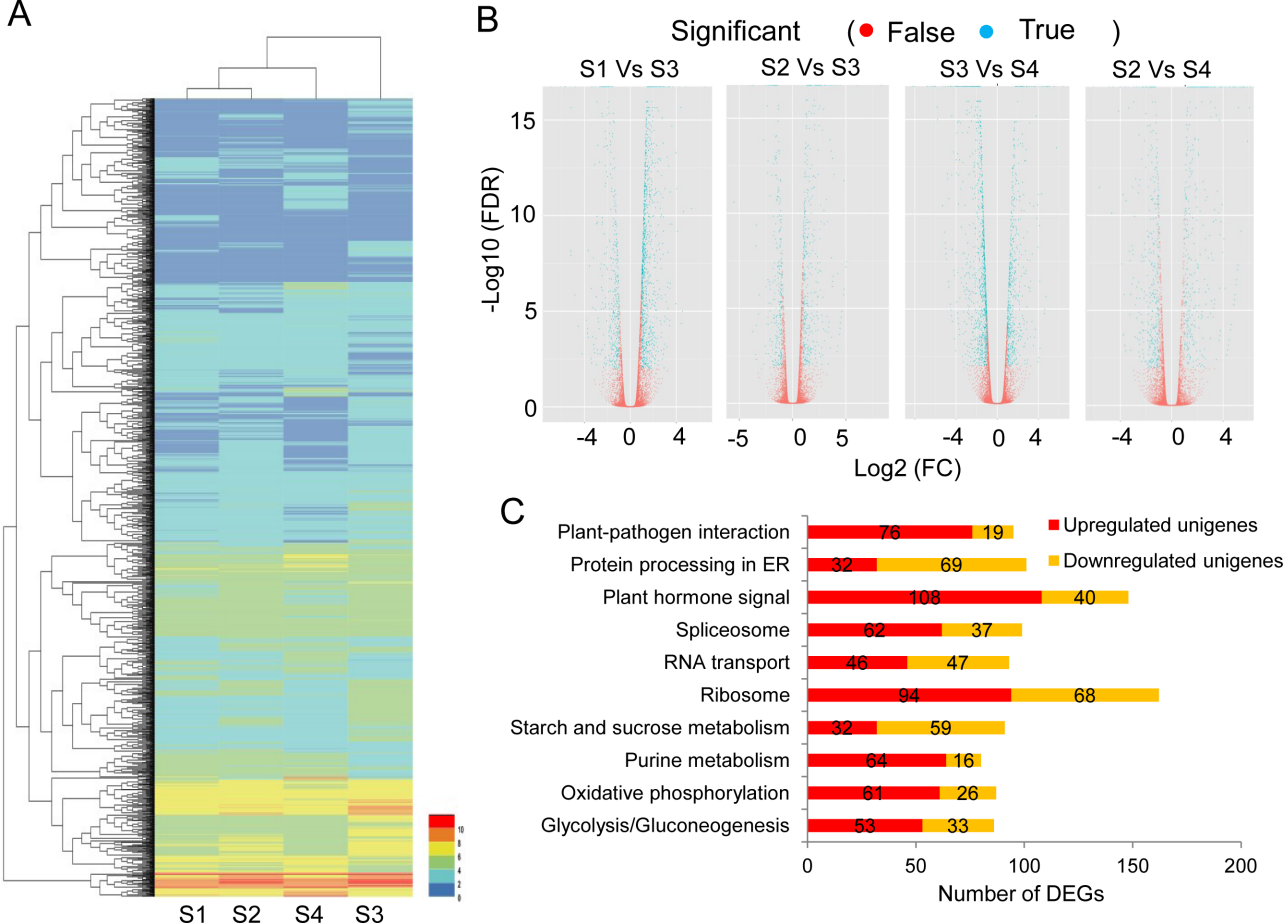
Hierarchical cluster and analysis for putative DEGs between both of node regions. (A), a heat-map profile of various families with different expression characteristics; (B), FDR analysis between both of node regions; (C), high transcripts of DEGs involved into top-10 hits of different metabolism pathways.

To assay the normality of RNA-seq data in four DEG libraries, we calculated the distribution of unique reads in each DEG library. The distribution over different reads abundance categories showed a similar pattern among four libraries (Table 1). Results from three biological replicates were highly similar (S2 Fig), suggesting a good reproducibility of the RNA-seq method. Additionally, the unigene expressions were also detected by the uniquely mapped DEG reads. A total of 2,818 unigenes were referred to as DEGs and used for the subsequent analysis (S3 Table). Hierarchical cluster demonstrated that DEGs were clustered into different groups (Fig 3A). A large number of DEGs were revealed by comparing the distribution between both of node regions (Fig 3B). In S1 vs S2, a total of 494 DEGs were found during Antirrhinum phyllotactic development, including 354 upregulated transcripts and 140 downregulated transcripts (S4 Table). A total of 1,479 unigenes were differently expressed in S1 vs S3, with 1,053 up-regulated transcripts and 396 downregulated transcripts (S5 Table). There were 154 up-regulated transcripts and 315 downregulated transcripts in S1 vs S4 (S6 Table). In S2 vs S3, 414 transcripts were upregulated and 280 transcripts were downregulated (S7 Table). By contrast, a total of 686 DEGs were demonstrated in S2 vs S4, with 332 upregulated transcripts and 354 downregulated unigenes (S8 Table). There was notable that 1,665 transcripts had different expressions with 550 upregulated DEGs and 1,115 downregulated DEGs in S3 vs S4 (S9 Table).

The rich KEGG analysis indicated that 2,818 DEGs were mapped into 118 signal pathways (S10 Table). The maps with the high unigene number were 162 unigenes in “ribosome pathway (ko03010)”, followed by 148 unigenes in “plant hormone signal transduction (ko04075)”, 101 unigenes in “protein processing in endoplasmic reticulum (ko04141)”, 99 unigenes in “spliceosome pathway (ko03040)”, 95 unigenes in “plant pathogen interaction (ko04626)” and 93 unigenes in “RNA transport (ko03013)” (Fig 3C). Especially, some unigenes involved in “starch and sugar metabolism” and “Glycolysis/Gluconegenesis” were upregulated from S1 to S4, while other groups showed the opposite expression patterns, such as “Regulation of autophagy”, “Biosynthesis of unsaturated fatty acids”, “Flavone and flavonol biosynthesis” and “Nitrogen metabolism”.

**Fig 4:**
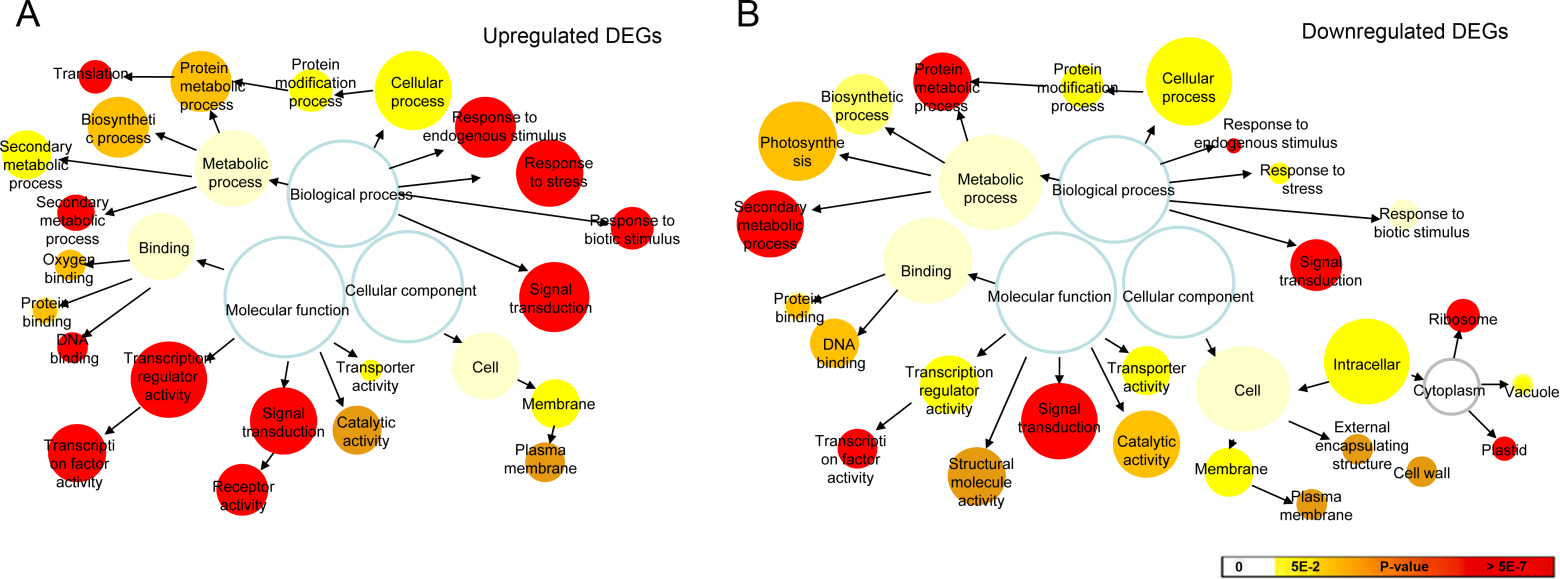
**GO terms enriched for the upregulated (A) and downregulated (B) DEGs.** The network graphs of the overrepresented GO terms for the combined clusters of DEGs. Colored nodes represented GO terms that were significantly overrepresented. The colors were shaded according to the significance level as shown in the color bar.

The DEGs were further subjected to the GO-term enrichment analysis and were classified into “cellular component”, “biological process” and “molecular function” (Fig 4). Within the cellular component category, the downregulated DEGs were significantly enriched in “cellular components” such as ribosome, plastid, vacuole, membrane, cell wall and extracellular region. The membrane components were mainly involved in the unregulated DEGs. Under the biological process, there were significant enrichment groups like “protein metabolism and modification processes”, “biosynthetic process”, “secondary metabolic process” as well as “response to biotic and abiotic stimulus”. Especially, a significant term focused on “response to endogenous or exogenous stimulus”. Under the molecular function category, most of DEGs were classified into “protein binding”, “transcription regulation”, “catalytic activity”, “transporter activity” and “signal transduction”.

### A complex regulation network of the plant-hormone pathways during the phyllotactic development

**Fig 5:**
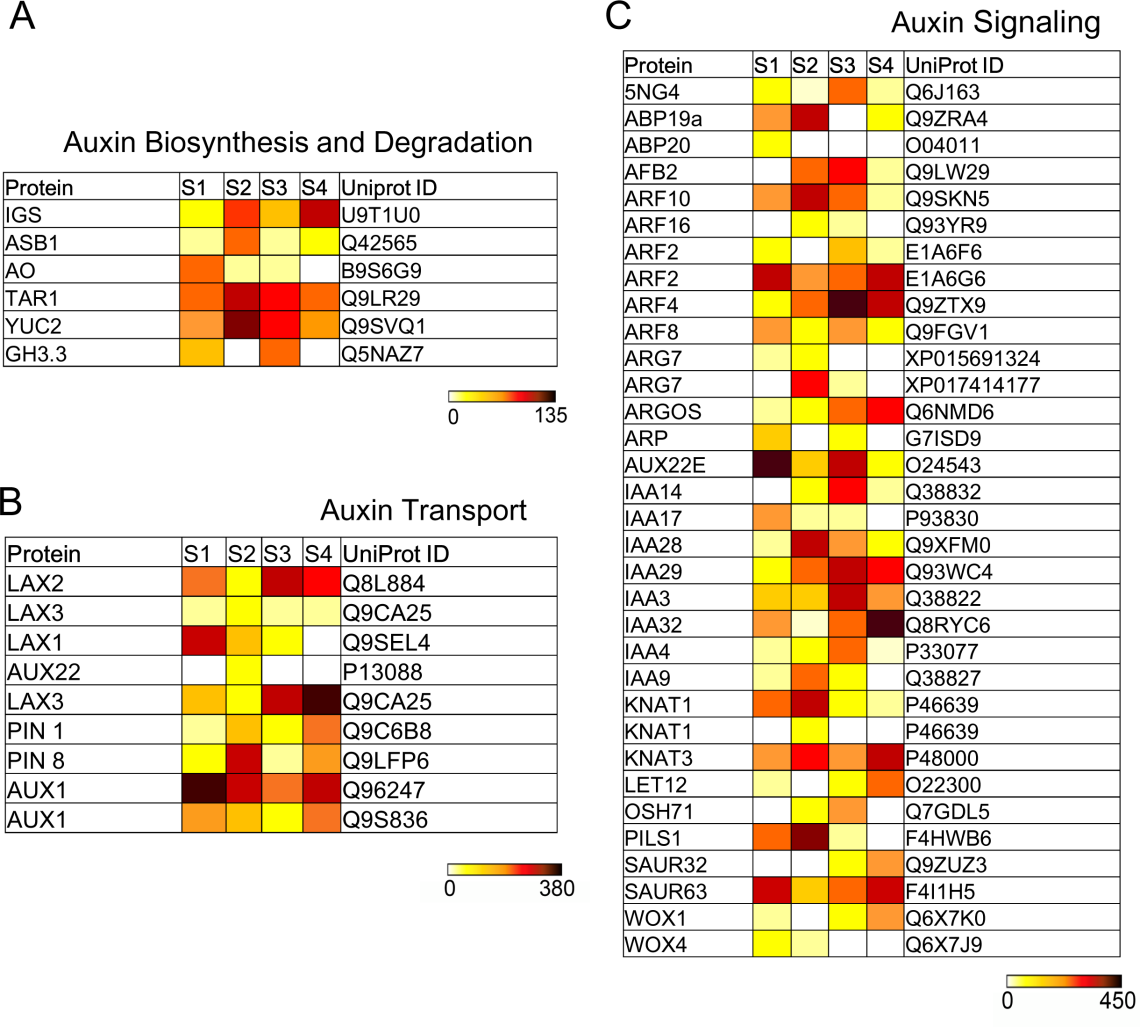
Heat maps presenting absolute expression values (RPM, reads per million reads mapped) for auxin-related genes related to auxin biosynthesis, transport and signaling. S1-S4 represented different node regions of Antirrhinum phyllotaxy, respectively. UniProt IDs were shown in the last lane of each table.

Based on the genome-wide transcript analysis, 89 DEGs were mapped into six hormone-related pathways in the Arabidopsis Hormone Database, including auxin, gibberellin, cytokinine, abscisic acid, ethylene and brassinosteroid. The largest group with differential expressions was related to auxin signal pathway. In auxin biosynthesis (Fig 5A), the identified regulators were the homologs of indole-3-acetaldehyde oxidase (AO), anthranilate synthase beta subunit (ASB), indole glucosinolates (IGS) and tryptophan aminotransferase related (TAR). The increases in the transcripts of *IGS* and *TAR1* were observed from S1 to S4, while *AO* and *ASB1* had higher expression levels in S1 and S2, respectively. The families of auxin influx-associated protein (AUX), auxin efflux carrier component (PIN) and AUX-like (LAX) were vital components in regulating auxin transport (Fig 5B). Both of AUX1 homologs demonstrated much higher expression levels in S1 and S4 than in S2 and S3. *AUX22* was only detected in S2. By contrast, *PIN1* has a high transcript in S4 and *PIN8* was observed in all node positions, particularly in S2. The high expression levels of *LAX 2*,*3* were found in S3 and S4. Additionally, other unigenes were also detected in auxin signaling (Fig 5C), including auxin response factor (ARF), indole-3-acetic acid (IAA), small auxin upregulated RNA (SAUR), glycoside hydrolase family 3 (GH3), auxin signaling F-box 2 (AFB2), auxin binding protein (ABP), homeobox protein knotted-1-like (KNAT, LET, OSH), WUSCHEL-related homeobox (WOX), indole-3-pyruvate monooxygenase (YUC), auxin-regulated gene controlling organ size (ARGOS), auxin-induced protein (5NG4) and indole-3-acetic acid induced protein (ARG). *ARF* and *IAA* in S3 presented much higher expression levels than those in other node regions.

**Fig 6:**
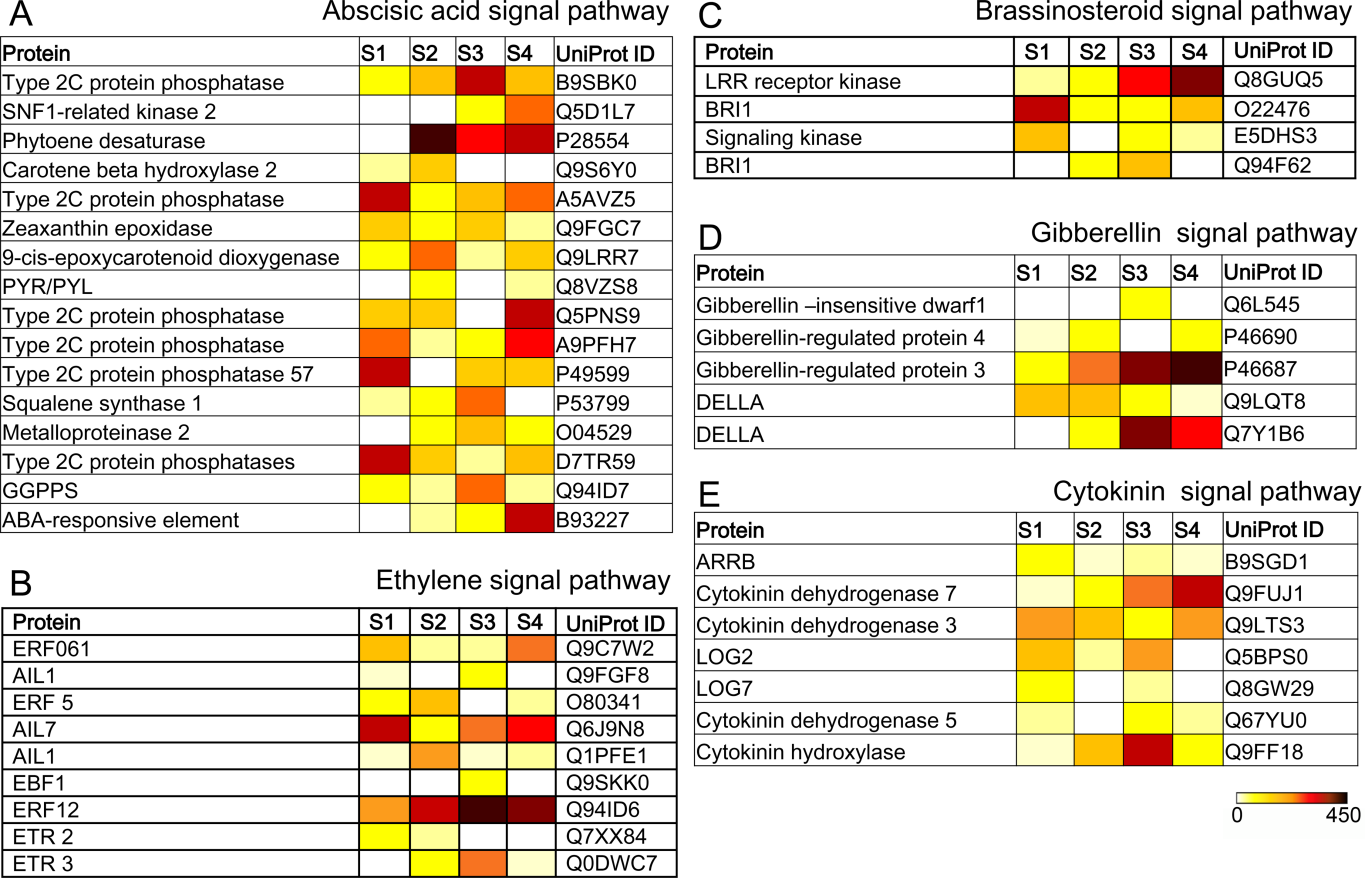
Heat maps showing absolute expression values for plant-hormone genes. S1-S4 indicates different stages of phyllotactic patterns, respectively. UniProt IDs were shown in the last lane of tables.

The DEGs were also observed in the abscisic acid, ethylene, brassinosteroids, gibberellin and cytokinin signal pathways. Total of 16 DEGs were shown in the abscisic acid signal pathway (Fig 6A), containing six type 2C protein phosphatases (PP2Cs), one SNF1-related kinase 2 (SnRK2), one phytoene desaturase (PDS), one carotene beta hydroxylase 2, one zeaxanthin epoxidase (ZEP), one 9-cis-epoxycarotenoid dioxygenase, one pyrabactin resistance/pyrabactin resistance like (PYR/PYL), one squalene synthase 1, one metalloproteinase 2 (MMP2), one geranylgeranyl pyrophosphate synthase (GGPPS) and one ABA-responsive element binding factor (ABF). In addition, nine DEGs were also clustered in the ethylene signal pathway (Fig 6B), including two ethylene receptors (ETRs), one encoding ein3-binding F-box protein (EBF), three AP2-like ethylene-responsive transcription factors (AILs) and three encoding ethylene-responsive transcription factors (ERFs). By contrast, one brassinosteroid LRR receptor kinase, two brassinosteroid insensitive 1 (BRI1) and one brassinosteroid signaling kinase were also shown in the brassinosteroid signal pathway (Fig 6C). Five unigenes such as one gibberellin-insensitive dwarf1 (GID1), two gibberellin-regulated proteins and two DELLA proteins were detected in the gibberellin signal pathway (Fig 6D), where *GID1* had much higher expression levels in S3 than that in other regions. In the cytokinin signal pathway, one specific type B ARR transcription factors (ARRB), two cytokinin riboside 5'-monophosphate phosphoribohydrolase (LOG), one cytokinin hydroxylase (CYP) and three cytokinin dehydrogenases (CKX) were found (Fig 6E). Therefore, most of DEGs involved in the hormone signal pathways were induced during swapping from vegetative to reproductive phase.

### Cluster of DEGs illustrated in the starch and sugar metabolic siganl pathways

**Fig 7:**
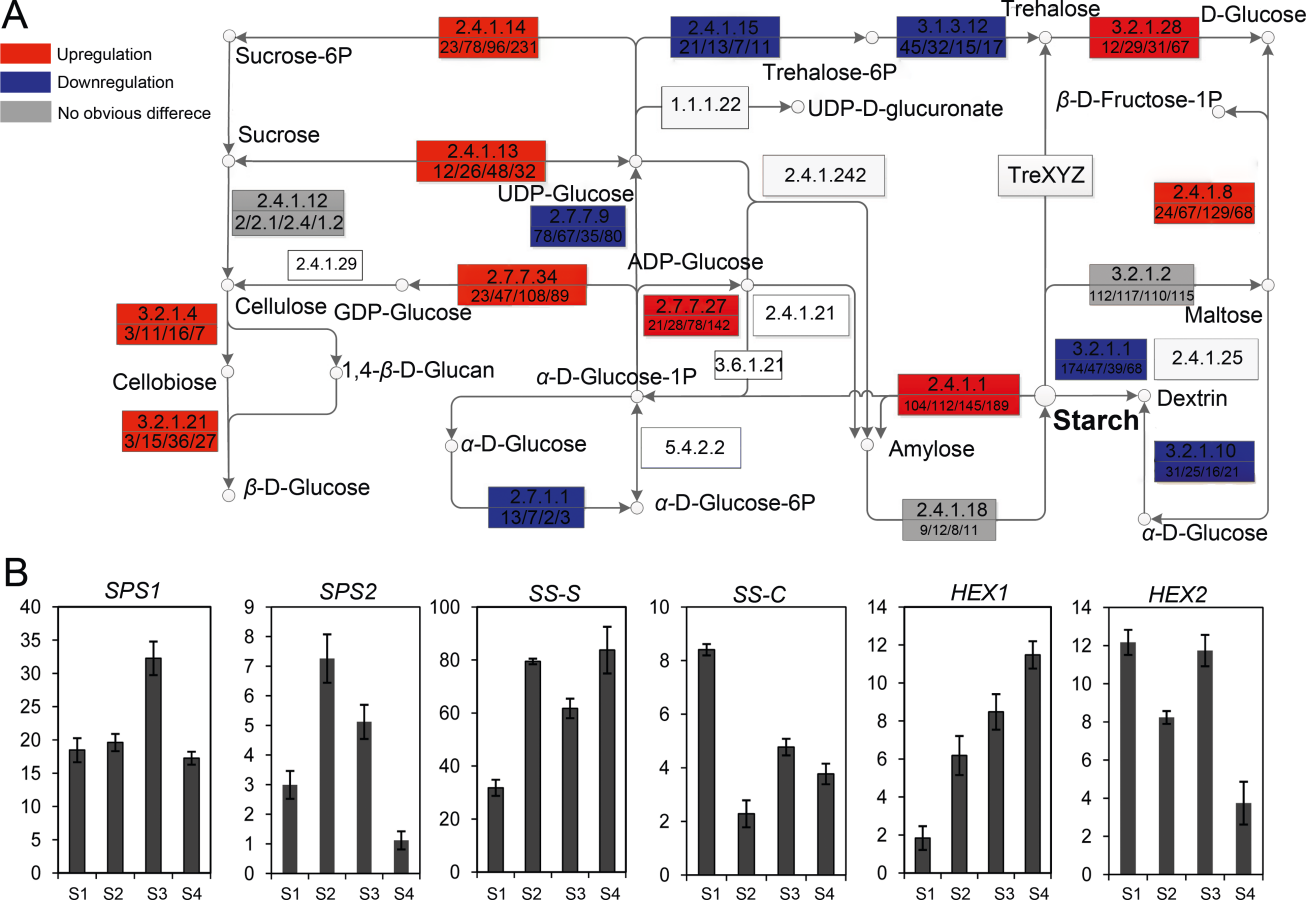
Expression profiles of carbohydrate metabolism-related transcripts in the simplified starch and sucrose metabolism pathways. A, The transcript of each gene was calculated and normalized based on RPM value. The red and blue boxes indicated the up-regulated and down-regulated enzymes, respectively. The gray boxes meant no significant transcription difference (*p* < 0.05, n = 3) and the white boxes represented these enzymes not detected in this study. The digitals in the upper or lower half of boxes were the EC numbers and the expression levels of unigenes from S1 to S4, respectively. B, The transcript levels of the selected unigenes related to the carbohydrate metabolism using qRT-PCR. SPS, Sucrose Phosphate Synthase; SS-S, Sucrose Synthase-Synthesis; SS-C, Sucrose Synthase-Cleavage; HEX, hexokinase.

To screen the carbohydrate-metabolism genes correlated with the phyllotactic establishment, DEGs were clustered by the MultiExperiment Viewer (MeV, v4.7.4) in the light of the K-means method and the hierarchical cluster, respectively (Fig 7A). By contrast, the transcripts encoding GDP-glucose pyrophosphorylase (EC: 2.7.7.34), endoglucanase (EC: 3.2.1.4) and *β*-D-glucosidase (EC: 3.2.1.21) showed much higher expression levels in S3 than these in other positions. The gene encoding ADP-glucose pyrophosphorylase (EC: 2.7.7.27) exhibited an expression level of 21 RPM in S1 and increased to 142 RPM in S4. There were no significant changes (*p* = 0.345) observed for the transcript of unigene encoding cellulose synthase (EC: 2.4.1.12). In the starch degradation branches, the expression level of transcript encoding trehalose-6-phosphate synthase (EC: 2.4.1.15) was down-regulated from 45 to 17 RPM. Additionally, carbohydrate metabolic branches that compete with the synthesis of starch were also measured, including hexokinase (EC: 2.7.1.1, HEX), sucrose phosphate synthase (EC: 2.4.1.14, SPS), and sucrose synthase (EC: 2.4.1.13, SS). Specifically, the transcripts of *SPS* and *SS* were significantly up-regulated (*p* < 0.001) at S4 and S3, respectively. No significant increase was detected for the expression levels of transcript-encoding starch-branching enzyme (EC: 2.4.1.18, *p* = 0.667) and *β*-amylase (EC: 3.2.1.2, *p* = 0.783). However, other transcripts encoding α-amylase (EC: 3.2.1.1), dextrin 6-glucano-hydrolase (EC: 3.2.1.10), trehalose-6-phosphate synthase (EC: 2.4.1.15), trehalose 6-phosphate phosphatase (EC: 3.1.3.12) and UDP-glucose pyrophosphorylase (EC: 2.7.7.9) exhibited lower expression levels at S3 or S4.

Several key genes encoding SPS, SS and hexokinase were selected for further analysis by qRT-PCR (Fig 7B). The expression characteristics were generated to illustrate the dynamic changes at different phyllotactic patterns. The enhanced expression levels of *SPS1* indicated that soluble sugars were produced via synthesis of sucrose, followed by hydrolysis into glucose and fructose. High *SS-S* expression levels implied that sucrose accumulation was a dominated process. Likewise, the upregualted *HEX* genes were also observed during the phyllotactic swapping.

### TF families involved in the establishment of phyllotactic morphology

**Fig 8:**
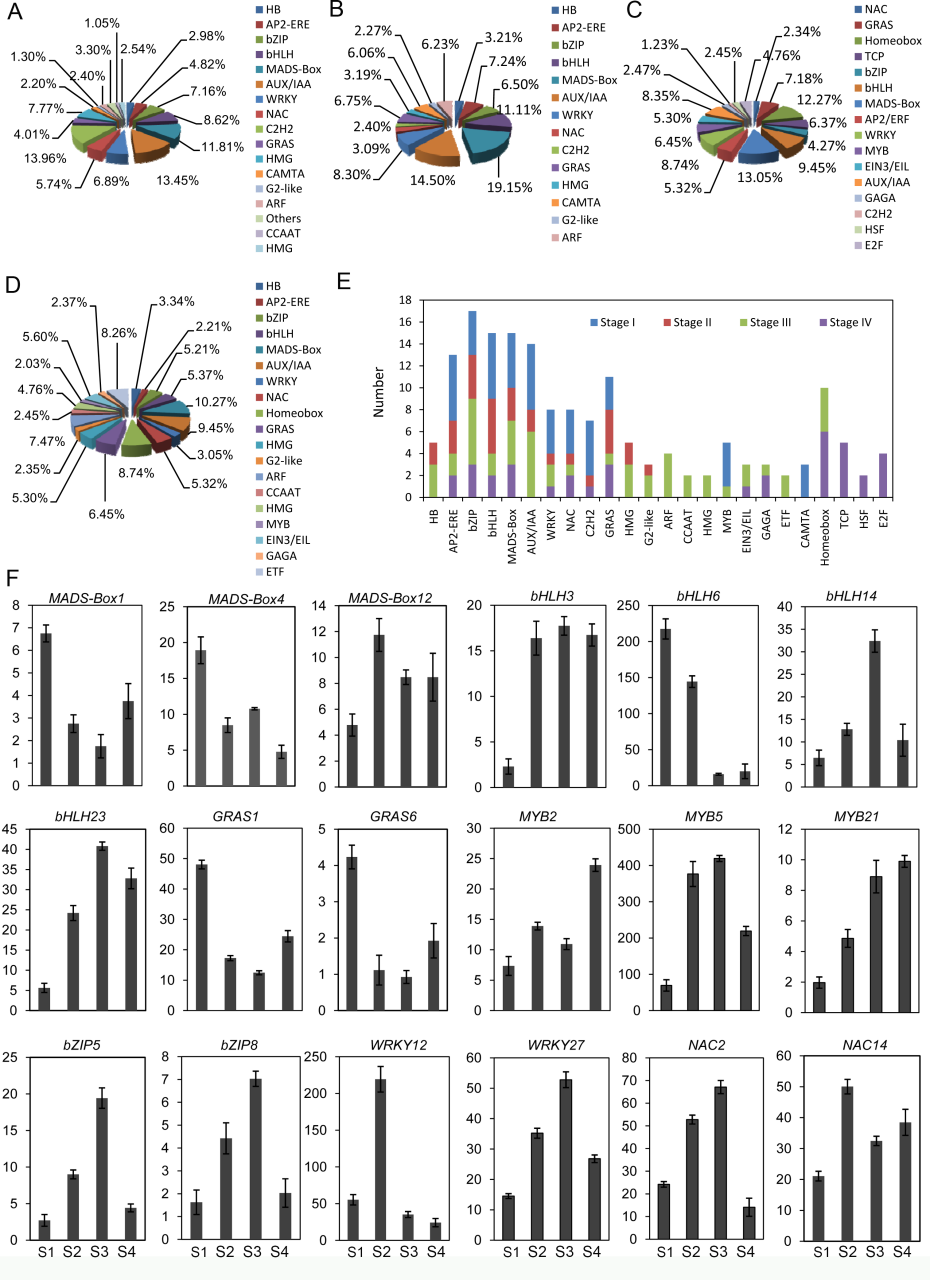
Distribution and expression profiles of Antirrhinum TFs at different node regions. (A) Distribution and type of TFs in S1; (B) Distribution and type of the TFs in S2; (C) Distribution and type of TFs in S3; (D) Distribution and type of TFs in S4; (E) Distribution and type of the differentially expressed TFs in different nodes; (E) The transcript profiles of the selected TFs investigated by qRT-PCR. Each point was the mean of three determinations. Vertical bars represented the standard error of the mean (n = 3).

A total of 179 transcripts putatively encoding TFs were differentially expressed during Antirrhinum phyllotaxy swapping. They were classified into 25 families, including APETALA2/ethylene responsive factor (AP2-ERE), basic region/leucine zipper motif (bZIP), basic helix-loop-helix (bHLH), ethylene-insensitive 3 and EIN3-like (EIN3/EIL), teosinte branched1/cycloidia/pcf (TCP), cup-shaped cotyledon (NAC), auxin/indole-3-acetic acid (AUX/IAA), auxin response factor (ARF), hot shock factor (HSF), WRKY, C2H2, GRAS, HB, HMG, G2-like, CCAAT, HMG, MYB, GAGA, ETF, CAMTA, Homeobox, E2F, and MADS-Box. By contrast, MADS-box, Homeobox, bZIP, AP2/ERF, AUX/IAA, bHLH, WRKY, EIN3/EIL and NAC family were the major members (Fig 8A–8C). The number and type of markedly increased in S3 and S4 (Fig 8D). Our qRT-PCR analysis revealed the transcription profiles of TFs, including three *MADS-boxe*s, four *bHLH*s, two *GRAS*s, three *MYB*s, two *bZIP*s, two *WRKY*s and two *NAC*s (Fig 8E). *MADS-box1* and *MADS-box4* exhibited a high expression level in S1, while the transcript peak of *MADS-box12* was detected in S2. Like results were observed in the expression profiles of *bHLH3*, *bHLH6*, *bHLH14*, and *bHLH23*. However, *MYB* genes (*MYB2*, *MYB5* and *MYB21*) mainly expressed in the spiral phyllotaxy. The significantly differential expressions were also observed in *bZIP*5, *bZIP8*, *WRKY12*, *WRKY27*, *NAC2*, and *NAC14*, when the phyllotactic swapping was happened.

## Discussion

With the development of Antirrhinum phyllotaxy, several signal pathways regulate dynamically cell components in different organ regions, resulting in the vegetative-reproductive transition. Accordingly, the ratio changes from opossite decussate to spiral phyllotaxy were detected (Fig 1). The phyllotactic development initiates by recruitment of cells from the peripheral region of SAM [3,39]. Auxin distribution is crucial for both recognizing the position of phyllotactic inception and facilitating organogenesis [20–25]. In our RNA-Seq data, auxin transporters such as PIN, AUX and LAX were differentially expressed from vegetative to reproductive phase during Antirrhinum phyllotactic development (Fig 5). The previous studies indicated that organ initiation was determined by the intracellular polarization of PIN and auxin accumulation [21–24]. Phyllotactic alteration in Arabidopsis was also shown in quadruple loss-of-function mutants of AUXs [23]. AUX exhibited a similar expression pattern with an auxin flux, which was located at the incipient site of phyllotactic formation [24]. Moreover, AUX and LAX might stabilize the PIN1-mediated auxin distribution and maximum [23]. For example, auxin stimulated the sharp expression patterns of PIN3 and LAX3 and the variations were observed in both the tissue geometry and the magnitude of auxin source [25]. The feedback system regulates an auxin concentration gradient across cell, promoting asymmetrical localization of these transporters [20–24], and resulting in the establishment of specific Antirrhinum phyllotaxy morphology.

Although the synthetic auxin with different transport properties induces organogenesis, the organ development and formation are not accurately positioned [25]. It means that additional regulators must be required to the developmental context for phyllotactic pattern formation. In our study, GH3s, ARFs, and AUX/IAAs were identified to participate in the regulation of Antirrhinum phyllotaxy alteration (Fig 5). Each member of these families was up-regulated rapidly in response to auxin addition [40–42]. GH3 can directly combine auxin to amino acids and maintain the maximum of auxin level, which can activate downstream reactions through the specific TIR1 receptor and the synergetic effect of ARF and AUX/IAAs [41–43]. The superfluous auxin increases the affinity of TIR1/F-box protein with AUX/IAAs [44]. High auxin started the degradation of AUX/IAA by the ubiquitin-proteasome pathway and the release of ARFs, promoting both the transcripts of AUX target genes and the hormone response [40–44]. Additionally, the homologs of ABP1, ARGOS, WOX, ARP and KNOX families were also detected. A loss-of-function mutant of *ABP1* generated an embryonic lethal phenotype [45]. Currently, the contradictory results showed that *ABP1* is not required for either auxin signaling or Arabidopsis development [46]. The reasons for the differences still need to be further elucidated. It was noticeable that auxin inhibited endocytosis of PINs by binding to ABP1 at the plasma membrane [45]. The plant overexpressing *ARGOS* was sensitive to auxin, implying its role for ubiquitous growth regulator during the establishment of phyllotactic morphology [47]. *WOX* was required for the adaxial/abaxial polarity establishment of the leaf margin development [48]. *ARPs* specifically expressed in lateral organ cells regulated the transcripts of *KNOXs* such as *KNAT1* and *KNAT2* [49]. Mutations of *ARPs* resulted in the determinacy loss of cell fate caused by ectopic expression of *KNOX1* [45,49].

Our transcriptome results also revealed that other hormone signal pathways like cytokinin, brassinosteroid, gibberellins, ethylene and abscisic acid were also involved into the control of Antirrhinum phyllotaxy morphology [35]. The importance of cytokinin signal for the regulation of maize phyllotaxy was identified by analysis of *abph1*. A loss-of-function mutant of *abph1* exhibited the opposite decussate phyllotaxy instead of the spiral phyllotaxy [50]. Cytokinin reduces the relative elongation rate and blocks the increase of meristem size [51]. Mutants for the cytokinin inhibitor *Arabidopsis* histidine phosphotransferase protein 6 (AHP6) have phyllotactic defects that affect the order in phyllotaxis [52]. In the brassinosteroid signal pathway, the BRI1 and its homologous genes were differently expressed in different phyllotaxy nodes of Antirrhinum (Fig 6). High brassinosteroid level in the boundary domain is disadvantage for organ separation, while the reduced level leads to the groove formation between the meristem and the new organ [53]. Root-specific brassinosteroid-deficiency in brevis radix/(brx) mutant causes reduced root growth due to reduction in the meristem size, and mature cell size as well [53]. Interestingly, brassinosteroids and auxin share downstream target genes, implying that the significant hormone crosstalk is vital for the phyllotaxtic development [51]. Similarly, gibberellin also regulates negatively phyllotactic complexity through GID1 and *DELLA* proteins identified in our libraries. In mutants, the increase of gibberellin level promotes the leaves prematurity and simple [54]. A declining gibberellin results in the spatial gradients of *DELLA* mRNA and protein abundance, regulating the phyllotaxtic development [55].

Although ethylene is deeply elaborated in the adjustment of seed germination, cell elongation, organ senescence and fruit development [56], a majority of regulators such as ERF, ETR, AIL and EBF was also differentially expressed in the different phyllotaxy nodes. ETRs first perceive the ethylene signal and inhibit the kinase activity of Constitutive Triple 1 (CTR1), which dephosphorylates the positive regulator Ethylene Insensitive 2 (EIN2). EIN3 triggers the ethylene responses by binding to ERF1/2 [57,58]. In addition, PYR/PYL, PP2C, and SnRK2 in the abscisic acid signal pathway were also associated with the establishment of phyllotaxtic morphology. Abscisic acid directly binds to the intracellular receptors PYR/PYL either as a monomer or as a dimer, promoting the formation of complexes involving PYR, PYL and PP2C proteins [59, 60]. The released SnRK2 from the complex of PP2C was activated by autophosphorylation [61]. The activated SnRK2 phosphorylates the abscisic acid responsive TFs (such as ABFs), regulating gene transcription [59].

Based on the crosstalk of signal pathways, these hormones can not only regulate many biological processes independently, but also cooperate between both of them. The synergistic action of Auxin and brassinosteroid was the first model to consider hormone signal integration during shoot vascular development [62]. The responsive was integrated to the common responsibility of cell elongation, where BIN2 inhibited ARF2 activity by the phosphorylation [15]. In addition, gibberellin and cytokinin promote the transcripts of *Aux/IAAs* and reduces the expression of *PIN* [63], whereas auxin increases the repressor transcriptions in the cytokinin signal pathway [64]. The peptide polaris (PLS) links auxin, ethylene and cytokinin hormone pathways [64,65]. PLS positively regulates auxin homeostasis by the inhibition of the ethylene and cytokinin responses. High auxin level activates the transcripts of the cytokinin signal inhibitor *AHP6* [52]. Additionally, ARR7/ARR15 transcriptions are considered as a conserved nexus for the auxin and cytokinin signal transduction [64]. The balance between the auxin and cytokinin pathways is required to regulate aspects of root development and establishment and maintenance of meristem size [18–21,50]. Hence, our RNA-Seq data presents one mechanism that distinguishes the collaborative action of different hormone signals in Antirrhinum phyllotactic development.

Interestingly, multiple regulatory components in the sugar signal pathway were involved into the interconnection with abscisic acid, auxin, and ethylene signal transductions. Many plants accumulate substantial starch and sugar reserves in leaves to provide carbon and energy for development and growth. The role of sugars as signal molecules modulates a range of vital processes such as seed germination, phyllotactic differentiation and light response [66,67]. Genetic and phenotypic analyses of sugar signaling mutants had unraveled complex and extensive interactions between sugar and hormonal signal pathways [68]. However, it is unclear whether the interconnection of sugar and hormone pathways was exhibited in specific cell types or at specific developmental stages. To address the question, the DEGs were identified in the sugar and starch metabolism pathway. Their expression profiles is benefit for understanding in detail the mechanism by which sugar signals are transduced in each pathway and the nature of signal molecules that participate in these processes.

The major TF families were also found between vegetative and reproductive phases in Antirrhinum (Fig 8). They were directly associated to either the general SAM development (MYB, bHLH, and WRKY) or the specific hormone pathway (AP2-ERE, AUX-IAA, Homebox, MADS-box, TCP, and bZIP) [69–71]. The number of key transcriptional regulators involved in SAM maintenance participated in the regulation of the hormone pathways. Analysis of mutants and transgenic plants showed that the downregulated expressions of *KNOXI* accelerated phyllotactic differentiation and decreased phyllotactic complexity [14–16]. Other evidences suggested that the downregulation of *KNOX* expression in initiating phyllotactic primordia may require auxin [70]. *KNOX-*mediated exclusion of gibberellin biosynthesis from the meristem confined gibberellins activity to developing leaf primordia. In addition, the specification of phyllotactic initials needed the interactions between the ARP (Asymmetric Leaves 1 [AS1]/Roughsheath 2 [RS2]/Phantastica [PHAN]) family of MYB-domain proteins and KNOX proteins [20,71]. *ARP* transcription factors (such as *AS* or *PHAN*) were specifically expressed in leaf initials and were required to correct phyllotactic development through the repression of *KNOX*s in the Arabidopsis and Antirrhinum leaves [69].

Important advances had been also obtained in determining the function of the adaxial fate-promoting HD-ZIPIII. For example, adaxial identity in Arabidopsis was specified by the class III HD-ZIP family genes *PHABULOSA (PHB)*, *PHAVOLUTA (PHV)* and *REVOLUTA (REV)* [72]. Triple mutants of the *phb*, *phv* and *rev* did not establish phyllotactic adaxial identity [72]. Further studies on the regulation of *HD-ZIPIII* gene expression had shown that patterns of *HD-ZIPIII* gene expression may impart patterning information to the apex [73]. Additionally, TCP transcription factors like *CIN* in Antirrhinum regulated phyllotactic shape and surface curvature by monitoring cell maturation at the transition zone of growing phyllotaxy [4,5]. A recent study identified LIPOXYGENASE2 (LOX2), the closest homolog of CIN in Arabidopsis, was involved in jasmonic acid biosynthesis [74]. An indirect link between TCP proteins and gibberellic acid had been also identified in Arabidopsis and tomato [75]. Thus, TCP directly promoted the transcription of genes involved in hormone signaling by binding to these respective genomic regions. However, how these TFs control the phyllotactic pattern by affecting the hormone signaling still need to be further elucidated.

## Conclusions

This study investigated the transcriptome profiles of vegetative-reproductive transition of Antirrhinum using Illumina RNA-seq and DEG deep-sequencing technologies. A total of 58,509 unigenes were assembled and annotated using different nr, KEGG, COG, and GO terms. Based on the heat-map and qRT-PCR data, a large number of DEGs were involved in the complicated signal networks, especially shown in the hormone signaling pathway and the carbohydrate metabolism. Most TF families with the high expression levels controlled the phyllotactic pattern by affecting the hormone and sugar-metabolism signal pathways. These findings provide a platform for further functional genomic research on Antirrhinum and a reference for studying complicated metabolism and regulation network in phyllotactic development.

## Supporting information

S1 Fig Function annotation of the Antirrhinum transcriptome using GO terms. (TIF)

S2 Fig Principal component analysis of transcripts expressed at four ages during the vegetative-reproductive transition of Antirrhinum. The same colors represent three replicate s at each stage.
(TIF)

S1 Table Primer sequences used in this study. (XLS)

S2 Table All unigenes shown in four libraries of different node regions. (XLS)

S3 Table Expression profiles of DEGs involved in four libraries of different node regions. (XLS)

S4 Table DEGs annotated between T1 and T2. (XLS)

S5 Table DEGs annotated between T1 and T3. (XLS)

S6 Table DEGs annotated between T1 and T4. (XLS)

S7 Table DEGs annotated between T2 and T3. (XLS)

S8 Table DEGs annotated between T2 and T4. (XLS)

S9 Table DEGs annotated between T3 and T4. (XLS)

S10 Table DEGs among four libraries involved in 118 KEGG pathways. (XLS)
